# Methylation-driven gene DLL3 is a potential prognostic biomarker in ocular melanoma correlating with metastasis

**DOI:** 10.3389/fonc.2022.964902

**Published:** 2022-10-20

**Authors:** Ludi Yang, Gaoming Wang, Hanhan Shi, Shichong Jia, Jing Ruan, Ran Cui, Shengfang Ge

**Affiliations:** ^1^ Department of Ophthalmology, Ninth People’s Hospital, Shanghai JiaoTong University School of Medicine, Shanghai, China; ^2^ Shanghai Key Laboratory of Orbital Diseases and Ocular Oncology, Shanghai, China; ^3^ Department of Hepatopancreatobiliary Surgery, Shanghai East Hospital, School of Medicine, Tongji University, Shanghai, China; ^4^ Tianjin Eye Hospital, Tianjin Key Lab of Ophthalmology and Visual Science, Nankai University Affiliated Eye Hospital, Tianjin Eye Institute, Tianjin, China

**Keywords:** ocular melanoma, DLL3, methylation, metastasis, prognosis

## Abstract

**Background:**

Ocular melanoma is an aggressive malignancy with a high rate of metastasis and poor prognosis. Increasing evidence indicated that DNA methylation plays an important role in the occurrence and development of ocular melanoma. Hence, exploring new diagnostic and prognostic biomarkers at the genetic level may be beneficial to the prognosis of patients with ocular melanoma.

**Methods:**

We collected DNA methylation and gene expression profiles of human UM (uveal melanoma) and CM (conjunctival melanoma) samples from various datasets. We conducted differential methylation and expression analyses to screen the potential biomarkers. Correlation analysis was performed to investigate the relationships between the expression level of DLL3 (delta-like protein 3) and the methylation level of its corresponding CpGs. We explored the prognostic and diagnostic value of DLL3 in UM and CM. Functional annotation and GSEA (gene set enrichment analysis) were applied to get insight into the possible biological roles of DLL3. A cohort of 60 ocular melanoma patients as well as UM and CM cell lines were used to validate our findings in bioinformatic analyses.

**Results:**

We found that *DLL3* was a methylation-driven gene correlating with UM metastasis. The CpGs of *DLL3* are mainly located in the gene body and their methylation level positively correlated to DLL3 expression. Multivariate Cox regression analysis revealed that DLL3 was an independent protective factor for UM patients. High DLL3 expression significantly prolonged the overall survival and disease-free survival of UM patients. DLL3 also showed a promising power to distinguish CM from normal tissues. Functional annotation exhibited that DLL3 may suppress UM progression through modulating immune activities and down-regulating various signaling pathways. External datasets, biospecimens, and cell lines further validated the aberrant expression and prognostic role of DLL3 in ocular melanoma.

**Conclusion:**

Methylation-driven gene *DLL3* could serve as a new potential diagnostic and prognostic biomarker in ocular melanoma. Our findings may contribute to improving the clinical outcomes of patients with UM or CM.

## Introduction

Ocular melanoma is the 2nd common melanoma in adults and may originate intraocularly from the uvea or extraocular from the conjunctiva. Ocular melanoma is an aggressive malignancy with a high rate of metastasis and poor prognosis (1–4). Oncogenic mutations in G-protein subunits α q (*GNAQ*) and 11 (*GNA11*) have been described in 80% of uveal melanomas (UM) (5), which could activate YAP/TAZ pathway and lead to abnormal cellular proliferation. BAP1 deletion and chromosomal anomalies have been also identified in UM (6, 7). Compared with uveal melanoma, conjunctival melanoma (CM) presents distinct gene expression patterns (8). Several oncogenic factors have been found in CM, such as *BRAF*, *NRAS*, and *TERT* mutations. These mutations are distinct from the mutations found in UM. Currently, most therapies on these genetic deficiencies have modest efficacy in both UM and CM. Consequently, exploring the molecular mechanism of ocular melanoma and determining the prognostic factors at the genetic level for early diagnosis is particularly important.

Loss of epigenetic homeostasis plays an important role in tumorigenesis by disrupting the normal pattern of gene expression (9). DNA methylation is a common epigenetic modification that regulates gene expression (10). Hypermethylation of the promoter CpG island is a major factor in tumorigenesis by inhibiting tumor suppressor genes (11). Evidence has also been presented that gene body DNA methylation can promote gene expression (12). In recent years, more and more studies have shown that DNA methylation plays an important role in the occurrence and development of ocular melanoma (13, 14). For example, p^16INK4a^, an inhibitor of cyclin-dependent kinases 4 and 6, plays an important role in the pRB tumor-suppressor pathways (15). Inactivation of *p^16INK4a^* gene through promoter hypermethylation has been frequently observed in various cancers. In UM, the promoters of *p^16INK4a^* were hypermethylated accompanied by a decrease in *p^16INK4a^* expression (16). In terms of conjunctival melanoma, p^16INK4a^ seems to be a good marker to identify melanoma from nevi and PAMs (17). *RASSF1A*, a tumor suppressor gene, has been found to be downregulated in many cancers (18). Promoter methylation of *RASSF1A* and decreased *RASSF1A* expression were found in both UM and CM (19). *RASSF1* is a tumor suppressor gene that controls tumor growth by inhibiting the RAS pathway. *RASEF* gene promoter hypermethylation was detected in UM samples without *RASEF* expression (20) (21). Many other genes are implicated in the loss of epigenetic homeostasis in ocular melanoma, such as *TIMP3*, *MGMT*, *hTERT*, and *RARB* in UM and *APC*, *CDKN2A*, and *WIF1* in CM (19).

Methylations of DNA have gradually demonstrated their value in the diagnosis of ocular melanoma and have become a potential new diagnostic tumor marker. In this study, we analyzed methylation and transcriptome profiles to find prognostic indicators related to abnormal methylation in UM. Integrated analyses of multiple ocular melanoma datasets revealed that *DLL3* was a dysregulated methylation-driven gene in UM metastasis and could serve as a biomarker to predict the prognosis of patients with UM. Results from biospecimens and cell lines further confirmed the prognostic value and expression pattern of DLL3 in ocular melanoma. The findings may be beneficial to therapeutic customization and medical decision-making.

## Methods

### Data collection and preparation

The methylation (Illumina HumanMethylation450 BeadChip) and transcriptome (RNA-Seq) profiles of 80 UM tissues were obtained from The Cancer Genome Atlas (TCGA) database (TCGA-UVM, https://portal.gdc.cancer.gov/). The corresponding clinicopathological information of UM patients from TCGA was obtained from the cBioPortal website (22) (https://www.cbioportal.org/). Considering that there were no normal tissues in TCGA-UVM, we downloaded another dataset(GSE57362 (23)) containing only methylation (Illumina HumanMethylation450 BeadChip) profiles of 10 uvea tissues and 15 UM samples from the Gene Expression Omnibus (GEO, https://www.ncbi.nlm.nih.gov/geo/). Other 57 UM cases from GSE44295 (Zhang’s cohort, platform: Illumina HumanRef-8 v3.0 expression beadchip) were employed for validation. GSE143952 (Mikkelsen’s cohort, platform: NanoString Custom Panel) and GSE148387 (24) (platform: Illumina NextSeq 500) both consisting of gene expression profiles of 12 CM samples and 8 healthy conjunctiva tissues were also included in this study. Fragments per kilobase million (FPKM) values of UM samples downloaded from TCGA were normalized as transcripts per kilobase million (TPM) and subsequently transformed as log2(TPM+1). The gene expression level of samples from GEO was transformed by log2(x+1).

### Bioinformatics analysis

A total of 485,512 DNA methylation probes were obtained and the methylation data were analyzed with the R package “ChAMP” (25). Principal component analysis (PCA) was conducted to detect the differences between samples based on the methylation profiles. After removing the abnormal samples, differential methylation analysis was performed to identify the dysregulated CpG sites between UM samples and normal uvea tissues with the thresholds of |log2FoldChange| > 0.5 and adjust p-values (padj) < 1×10^-5^. The prognostic CpG sites were screened through univariate Cox regression analysis on the basis of UM patients from TCGA with the cut-off criteria of p-value < 0.05.

Differentially expressed genes (DEGs) between non-metastatic and metastatic UM samples were calculated based on the count matrix with the R package “DESeq2” (26). Those genes with |log2FoldChange| > 0.5 and padj < 0.05 were selected as metastasis-related genes. The possible biological function of DLL3 was annotated by Gene Ontology (GO) and Kyoto Encyclopedia of Genes and Genomes (KEGG) enrichment analyses through the R package “clusterProfiler” (27). Gene set enrichment analysis (GSEA) was conducted to explore the potential regulatory mechanisms of DLL3 with the annotated gene sets in “h.all.v7.4.symbols.gmt” as reference (28). The results of bioinformatics analyses were visualized utilizing the R packages “ggplot2”, “pheatmap”, “survminer”, and “VennDiagram”.

### Patient samples

60 human ocular melanoma tissues and 18 human normal melanocyte tissues were collected from patients in Ninth People’s Hospital, Shanghai Jiao Tong University School of Medicine from 2007 to 2017. Histological characteristics of all samples were assessed by pathologists according to standard criteria, and clinicopathological characteristics of patients with ocular melanoma are listed in **Table S1**.

### Cell lines

The PIG1 human normal melanocyte cell line was kindly provided by the Department of Ophthalmology, Peking University Third Hospital. Human retinal pigment epithelium cell line ARPE-19 was purchased from ATCC. Human UM cell lines OMM2.3, OMM1, MEL290 as well as the human conjunctival melanoma cell lines CRMM1, CRMM2, and CM2005.1 were kindly given by Prof. Martine J. Jager (Department of Ophthalmology, Leiden University Medical Center, The Netherlands). UM cell line 92.1 was kindly provided by Professor John F Marshall (Tumor Biology Laboratory, John Vane Science Centre, London, UK). All cell lines used in this study were authenticated with STR profiling.

### Cell culture

PIG1, ARPE-19, MEL290, OMM2.3, OMM1, 92.1 and CM2005.1 cells were cultured in RPMI 1640 medium (GIBCO). CRMM1 and CRMM2 cells were cultured in Ham’s F-12 K (Kaighn’s) Medium (GIBCO). All mediums were supplemented with 10% heat-inactivated fetal bovine serum (FBS; GIBCO), 100 U/ml penicillin, and 100 mg/ml streptomycin and all cells were cultured at 37 °C in a humidified 5% CO_2_ atmosphere.

### Immunofluorescence

Cells which are adhering to a glass slide were fixed with 4% formaldehyde (Fisher) for 15 min, then blocked with 5% normal goat serum (Vector) with 0.1% Triton X-100 in PBS for 60 min at room temperature. Immunostaining was performed by using the appropriate primary and secondary antibodies. Nuclei were counterstained with DAPI. IF staining was performed with the appropriate secondary antibody (Invitrogen, 1:1000 dilution). Images were taken with a ZEISS Axio Scope A1 Upright Microscope.

### Isolation of RNA and Quantitative Real-Time RT-PCR

Total RNA was extracted from samples using the EZpress RNA Purification Kit (B0004), and cDNA was generated using the PrimeScript RT Reagent Kit (Takara). Quantitative real-time PCR using Powerup SYBR Green PCR Master Mix (Life Technologies) was performed using a real-time PCR system (Applied Biosystems), with the following procedures: 95°C for 10 min, 40 cycles of 95°C for 15 s and 60°C for 1 min. The changes in the mRNA levels were quantified using GAPDH mRNA as the control. The primers used in this study were as follows: DLL3-F, 5′- GGGCAGCTGTAGTGAAACCT-3′; DLL3-R, 5′- CTTCACCGCCAACACACAAG-3′; GAPDH-F, 5′-GAGCTGAACGGGAAGCTCACTG-3′; GAPDH-R, 5′-TGGTGCTCAGTGTAGCCCAGGA-3′.

### Statistical analysis

The analyses were performed using GraphPad Prism 9 and R software (version 4.1.0). We applied Wilcoxon rank-sum test to analyze the difference in gene expression between two groups. The Kaplan–Meier method was applied to produce the survival curve and the difference between groups was compared with the log-rank test. Pearson correlation test was conducted to evaluate the relationship between two variables. Univariate and multivariate Cox regression analyses were employed to assess the prognostic value of factors. Receiver operating characteristic (ROC) curves and the area under the curve (AUC) values were introduced to evaluate the predictive performance. Statistical significance was set at p-value <0.05.

## Results

### DLL3 was a dysregulated methylation-driven gene in UM metastasis

**Figure 1:**
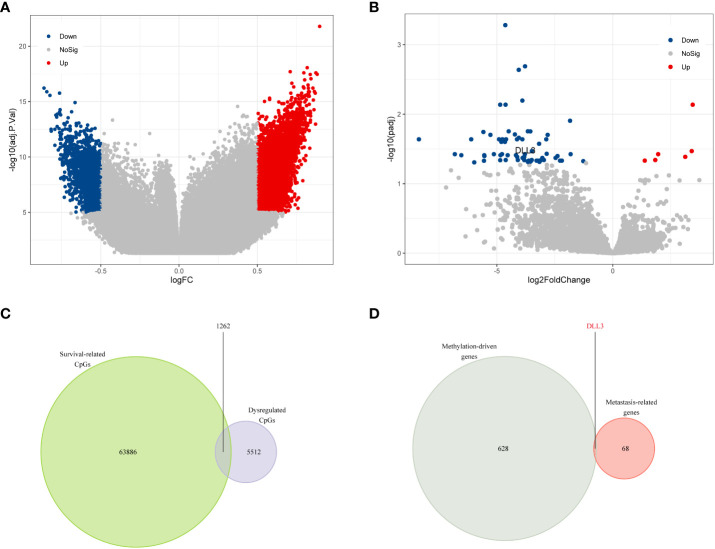
DLL3 was identified as a dysregulated methylation-driven gene that correlated with UM metastasis. **(A)** Volcano plot of differential methylation CpGs between UM samples and normal uvea tissues. **(B)** Volcano plot of DEGs between metastatic and non-metastatic UM samples. **(C)** Venn diagram to screen the dysregulated prognostic CpGs. **(D)** DLL3 was identified as a methylation-driven gene correlating with UM metastasis.

Since aberrant DNA methylation may contribute to tumor progression (29), we first analyzed the methylation data of 15 UM samples and 10 normal uvea tissues from GSE57362. The quality control step was first conducted to minimize the statistical error caused by abnormal samples. Although the UM and uvea tissues exhibited obvious dissimilarity (**Figure S1A**), there were still three samples not well clustered (**Figure S1B**). After removing those outlier samples, a total of 13 UM samples and 9 uvea tissues that showed distinct differences were left for the following analyses (**Figure S1C**). We subsequently performed differential methylation analysis to identify the dysregulated CpGs. Compared to normal uvea tissues, a total of 5,303 upregulated and 1,471 downregulated CpGs were screened in the UM samples (**Figure 1A**). To evaluate the prognostic value of CpGs, we utilized univariate Cox regression analysis based on the clinical and methylation data obtained from TCGA. Of 394,475 candidate CpGs, 65,148 CpGs were found to be significantly correlated with the OS (overall survival) of UM patients.

As metastasis is common in UM and seriously threatens the survival of UM patients, we tried to figure out the key genes correlating with metastasis. DEGs between non-metastatic and metastatic UM samples were calculated, and there were 6 upregulated and 63 downregulated genes screened in the metastatic samples compared to the non-metastatic ones (**Figure 1B** and **Table S2**). The correlation between the DEGs expression level and their DNA methylation level was also investigated in UM (**Table S3**). Taking an intersection for the survival-related CpGs and dysregulated CpGs, 1,262 overlappings were obtained and considered as survival-related dysregulated CpGs (**Figure 1C**). After annotation of these survival-related dysregulated CpGs, 629 methylation-driven genes were identified. Finally, *DLL3* was screened as the only methylation-driven gene of interest associated with UM metastasis (**Figure 1D** and **Table S4**).

### DLL3 served as a biomarker correlating with the prognosis of patients with UM

**Figure 2:**
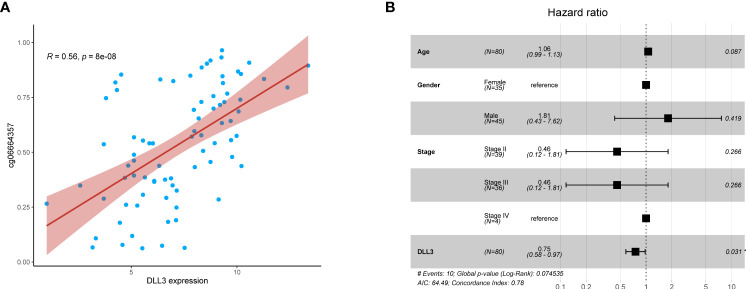
DLL3 expression was positively correlated with its DNA methylation level and served as an independent protective factor for UM patients. **(A)** The relationship between DLL3 expression and its methylation CpG cg06664357. **(B)** Multivariate Cox regression analysis indicated that DLL3 played a protective role in the prognosis of UM patients. *P < 0.05.

DNA methylation regulates gene expression and the function varies with the location of methylation sites (30). Existing evidence indicated that DNA methylation in promoters suppresses gene expression, while gene body methylation may stimulate expression (12). cg06664357 was one of the survival-related dysregulated CpGs and located in the gene body of *DLL3*. In this study, we analyzed the relationship between cg06664357 and *DLL3*, founding that *DLL3* expression increased with the methylation level of cg06664357 (R = 0.56, p < 0.001, **Figure 2A**). To determine whether it was an exceptional case, we also analyzed other CpGs located in the *DLL3* gene. As shown in **Figure S2**, *DLL3* expression was positively correlated with the methylation levels of most CpGs. Since most CpGs of *DLL3* belong to the gene body, it’s not surprising that the methylation of *DLL3* promoted its expression. Based on the relationship between DNA methylation and gene expression of *DLL3*, we speculated that *DLL3* was upregulated in UM tissues because all the CpGs of *DLL3* exhibited higher methylation levels compared to normal uvea.

**Figure 3:**
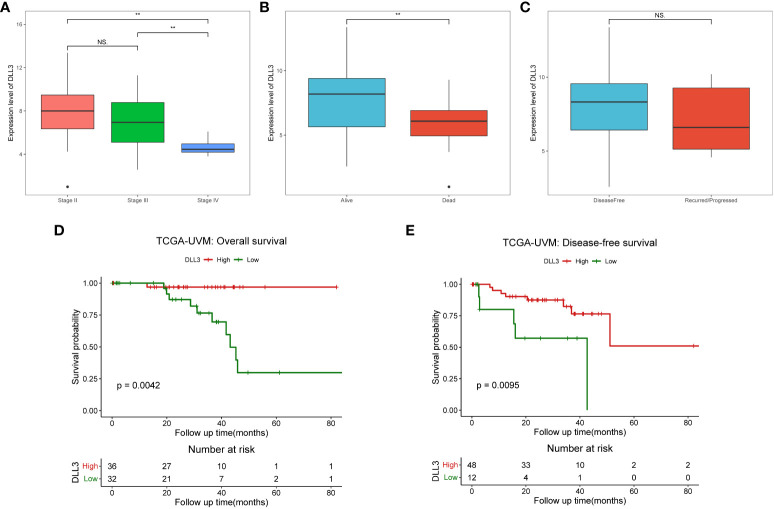
UM patients with high DLL3 expression were more likely to have favorable clinical outcomes. **(A–C)** The relationships between DLL3 expression and clinical features, including pathological stage **(A)**, living status **(B)**, and disease-free status **(C)**. **(D, E)** Kaplan-Meier curves demonstrated that high DLL3 expression significantly prolonged the overall survival **(D)** and disease-free survival **(E)** of UM patients. NS, no statistical significance, **P < 0.01.

To investigate the prognostic value of DLL3 in patients with UM, we employed multivariate Cox regression to analyze the connections between *DLL3* expression together with clinicopathological characteristics and OS. Depicted as a forest plot in **Figure 2B**, our results revealed that *DLL3* expression was an independent protective factor for the OS of patients with UM (p = 0.031). We subsequently explored the association between *DLL3* expression and various clinical features of UM patients. Increased expression level of DLL3 was negatively correlated with the pathological stage (**Figure 3A**). Besides, UM patients with higher expression level of DLL3 seemed to exhibit better clinical outcomes (**Figure 3B**). The mean level of DLL3 expression of UM samples in the disease-free group was higher than that in the progressed group although there was no statistical difference (**Figure 3C**). To further get insight into the prognostic significance of DLL3, we assigned the UM patients into high and low *DLL3* expression groups according to the optimal cutoff value. Kaplan-Meier curves demonstrated that UM patients in the high DLL3 expression group showed a longer survival time (p = 0.0042, **Figure 3D**). In addition, high *DLL3* expression also prolonged the disease-free survival (**Figure 3E**). Taken together, *DLL3* may serve as a prognostic biomarker for patients with UM.

### Exploration of the biological function of DLL3 in UM

**Figure 4:**
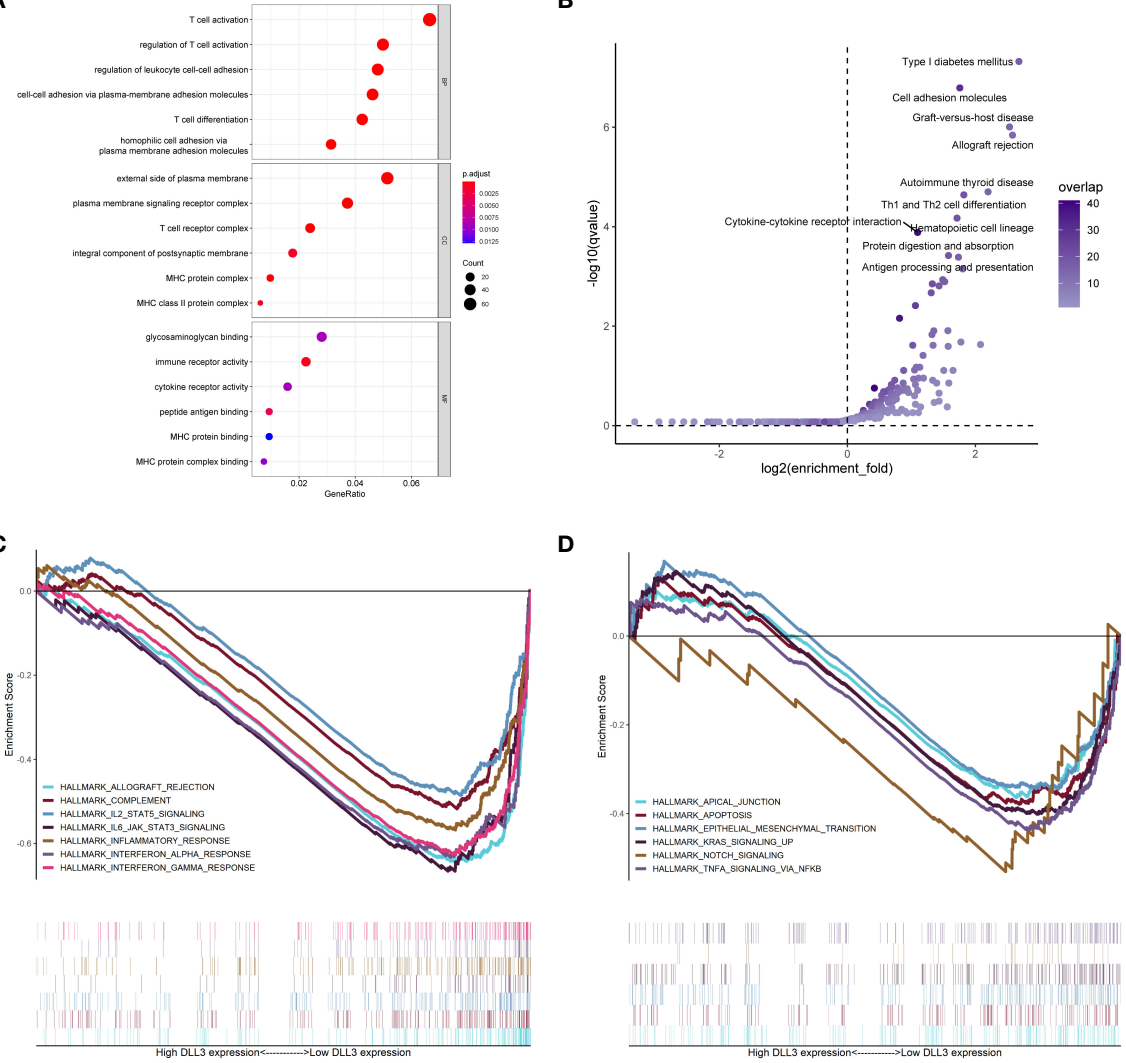
Functional annotation of DLL3 in UM. **(A, B)** GO **(A)** and KEGG **(B)** enrichment analyses of the DEGs based on DLL3 expression. BP, biological process; CC, cellular component; MF, molecular function. **(C, D)** GSEA was applied to explore the regulatory mechanisms of DLL3 in UM with the hallmarks of cancer set as the reference gene sets.

To get an insight into the potential regulatory mechanisms underlying DLL3, we divided UM patients into high and low *DLL3* expression groups according to the median level. Compared to the low *DLL3* expression group, a total of 1,978 DEGs consisting of 1,111 upregulated and 867 downregulated genes were screened (**Table S2**). GO enrichment analysis revealed that the DLL3-related DEGs mainly participated in immunoregulation and responses including “T cell activation”, “regulation of leukocyte cell-cell adhesion”, “immune receptor activity”, “peptide antigen binding”, and others (**Figure 4A** and **Table. S5**). Notably, at the cellular component (CC) levels, the enriched items such as “external side of plasma membrane” and “plasma membrane signaling receptor complex” indicated that DLL3 played an important role on the cell membrane which agrees with that DLL3 is a cell surface Notch ligand (31). KEGG annotation exhibited that pathways including “cytokine-cytokine receptor interaction”, “Th1 and Th2 cell differentiation”, “cell adhesion molecules”, and “antigen processing and presentation” were significantly enriched (**Figure 4B** and **Table S5**). In addition, GSEA algorithm and gene sets of cancer hallmarks were utilized to further explore the role of DLL3 in tumor progression. As displayed in **Figure 4C and 4D**, hallmarks and signaling pathways like “NOTCH signaling”, “IL2-STAT5 signaling”, “KRAS signaling”, “TNFα signaling *via* NF-κB”, “interferon-γ response”, “inflammatory response”, and “epithelial-mesenchymal transition” were significantly enriched in the low *DLL3* expression group and there were no obvious hallmarks found in the high *DLL3* expression group (**Table. S5**). Therefore, DLL3 may suppress tumor development and metastasis by regulating immune responses and inhibiting various signaling pathways.

### External and experimental validation of the prognostic value of DLL3 in UM and CM

**Figure 5:**
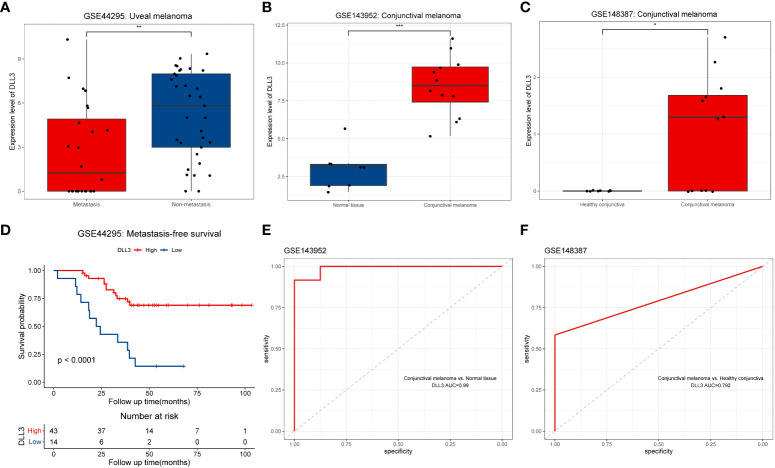
External validation of the prognostic value of *DLL3* expression in UM and investigation of the diagnostic performance in CM. **(A-C)** Boxplots to show the expression levels of DLL3 in ocular melanoma and normal tissues from three datasets including GSE44295 **(A)**, GSE143952 **(B)**, and GSE148387 **(C)**. **(D)** Kaplan-Meier curve indicated that UM patients with higher DLL3 expression have longer metastasis-free survival. **(E, F)** The ROC curves indicated that DLL3 expression showed a promising power to distinguish CM samples from normal tissues with an AUC value of 0.99 in GSE143952 **(E)** and 0.792 in GSE148387 **(F)**. *P < 0.05, **P < 0.01, ***P < 0.001.

To further validate the prognostic value of DLL3 in UM, we employed an external cohort consisting of 57 UM patients from GSE44295. The expression level of DLL3 in the non-metastatic UM samples was distinctly higher than that in the metastatic ones (**Figure 5A**). Kaplan-Meier survival curve demonstrated that UM patients with high DLL3 expression level exhibited longer metastasis-free survival than patients with low DLL3 expression level, highlighting the protective role of DLL3 in UM (**Figure 5D**). Considering the lack of UM cohorts with integrated data of gene expression and corresponding clinical information, we employed two external datasets containing a number of CM samples to test if DLL3 still worked well. As expected, the expression level of DLL3 in CM samples was significantly higher than that in the healthy conjunctiva tissues in both datasets (**Figures 5B, C**). In addition, DLL3 performed a promising power to distinguish CM samples from normal conjunctiva tissues in GSE143952 and GSE148387 with the AUC values of 0.99 and 0.792, respectively (**Figures 5E, F**).

### Experimental validation of the role of DLL3 using biospecimens

**Figure 6:**
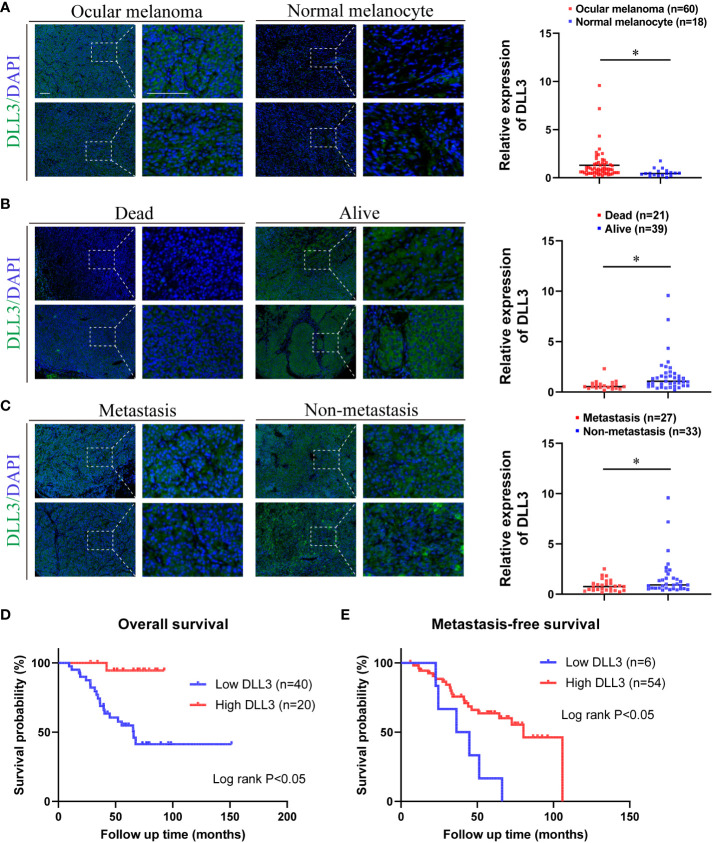
Ocular melanoma biospecimens were used to validate the aberrant expression and prognostic value of DLL3. **(A)** Representative immunofluorescence staining of DLL3 in ocular melanoma and normal melanocyte (left). Quantified data for DLL3 staining showed that the expression level of DLL3 in ocular melanoma samples was significantly high than that in normal melanocyte tissues (right). **(B)** Representative immunofluorescence staining of DLL3 in ocular melanoma samples from patients with different living statuses (left). Quantified data for DLL3 staining showed that the expression level of DLL3 in alive patients was significantly higher than that in patients who died during the follow-up (right). **(C)** Representative immunofluorescence staining of DLL3 in ocular melanoma patients with or without the metastasis occurrence (left). Quantified data for DLL3 staining showed that the expression level of DLL3 was higher in non-metastatic samples than that in metastatic ones (right). **(D, E)** Kaplan-Meier curves showed that high DLL3 expression significantly prolonged the overall survival **(D)** and metastasis-free survival **(E)** of patients with ocular melanoma. Scale bar = 50μm. *P < 0.05.

**Figure 7:**
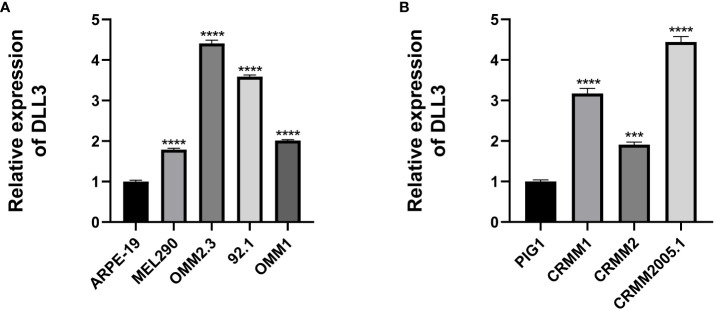
Cell lines were used to validate the aberrant expression of DLL3 in ocular melanoma. **(A)** The expression level of DLL3 in UM cell lines was significantly higher than that in the control cell line. **(B)** The expression level of DLL3 in CM cell lines was high compared to the control. ***P < 0.001, ****P < 0.0001.

Considering that the results above were based on public databases, we performed immunofluorescence using biospecimens to further verify the role of DLL3 in ocular melanoma. Of note, the expression of *DLL3* was upregulated in ocular melanoma tissues compared to normal melanocyte tissues (p < 0.05, **Figure 6A**). Besides, patients with a high expression level of DLL3 showed favorable clinical outcomes (**Figures 6B, C**). High *DLL3* expression prolonged the OS of patients with ocular melanoma (**Figure 6D**). Similarly, Kaplan-Meier curve indicated that patients with lower *DLL3* expression are more likely to present with metastasis than patients with higher expression of this gene (**Figure 6E**). Patients with higher *DLL3* expression have worse OS. Consistently, we also observed increased *DLL3* expression in ocular melanoma cell lines (**Figures 7A, B**). Taken together, DLL3 may serve as a diagnostic and prognostic biomarker in ocular melanoma.

## Discussion

Ocular melanoma, composed of UM and CM, is highly malignant with a poor prognosis. Ocular melanoma accounts for 4%-6% of melanomas with an increasing incidence. Almost 50% of ocular melanoma patients will develop metastasis within 10 years of treatments (32, 33). The clinical evaluation of ocular melanoma is fundamentally based on ophthalmoscopy, biomicroscopy, ultrasonography and radiography. MRI plays a significant role in disease evaluation (34). However, imaging examinations are hard to detect ocular melanoma at an early stage, as well as metastasis. Currently, the main treatment strategies for ocular melanoma include resection, adjuvant chemoradiation and eye enucleation (35, 36). Nonetheless, most patients survive less than 12 months after metastatic diagnosis, as there is no effective treatment for metastatic ocular melanoma. Therefore, it is of great significance to explore the potential metastatic-related markers and potential therapeutic targets for preventing early-stage metastasis and improving the prognosis (37). It has been demonstrated that the genetic high-risk features, such as chromosome 8q gain and loss of *BAP1*, are frequent in patients with metastatic disease (38). Recently, high levels of nestin expression have been shown to correlate with metastatic progression and reduced survival rate in UM patients (39). Furthermore, Histopathologic identification achieved with immunohistochemistry using CD31 and D2–40 antibodies provides histopathologic evidence of extravascular spread of a primary CM (40). Caltabiano R. et al. evaluated the expression of *ADAM10*, *RKIP* and *pRKIP* and showed them as negative prognostic markers in UM (41, 42). In addition, Broggi G et al. identified ABCB5 as a prognostic factor in UM and observed that higher ABCB5 immunohistochemical levels were associated with a higher risk of metastasis (43). Lally SE et al. evaluated genetic mutations and molecular genetic pathways in CM and reported that loss of *ATRX* and *ALT* may be early events in CM. They also confirmed that *NRAS* mutation implied an increased risk for metastasis and death in CM (44). Though mutations are frequently present in CM, little is known about prognostic genetic biomarkers due to the low incidence of this disease.

In this study, we found that *DLL3* was a dysregulated methylation-driven gene that correlated with UM metastasis. Based on the positive correlation between the methylation and expression of DLL3, we inferred that DLL3 was highly expressed in the UM samples, which was further validated by biospecimens and cell lines. We investigated the role of DLL3 in the prognosis of patients with UM, finding that DLL3 could serve as a protective factor. The relationships between DLL3 and clinical characteristics indicated that UM patients with high DLL3 expression tended to achieve favorable clinical outcomes, including earlier pathological stage, prolonged overall survival, and a lower possibility to get disease recurred or progressed. In addition, DLL3 exhibited a promising power to distinguish CM from healthy conjunctiva. Therefore, the methylation-driven gene *DLL3* may act as a diagnostic and prognostic biomarker in ocular melanoma.

The Notch signaling pathway is a highly conserved signaling pathway involved in a variety of development processes. *DLL3* is an inhibitory Notch pathway ligand, which mediates cell-fate decisions and is tumor-suppressive or oncogenic depending on the cellular context (45). In small cell lung cancer (SCLC), *DLL3* is highly expressed in more than 80% of patients and is highly expressed in both the cell membrane and cytoplasm of the tumor. Clinical studies have shown that the high expression of *DLL3* in SCLC is negatively correlated with patient survival (46). DLL3 is identified as a regulator factor of SCLC‐cell proliferation, migration, and invasion and an oncogene related to modulating Snail expression (45). In prostate cancer, *DLL3* is highly expressed in a subset of advanced metastatic samples and is not notably expressed in non-metastasis samples or benign tissues (47). An antibody-drug conjugate that targets DLL3 named Rovalpituzumab tesirine (SC16LD6.5) has complete and durable responses in DLL3-expressing prostate cancer (47). In other cancer types, gene silencing of *DLL3* induces intrinsic apoptosis, resulting in significant growth inhibition. For example, overexpression of *DLL3* induces cellular apoptosis in human hepatocellular carcinoma. *DLL3* expression is silenced during hepatocarcinogenesis in association with HBV infection *via* an epigenetic mechanism (48). For melanoma, knockdown of *DLL3* inhibits inflammatory stimulation-induced melanoma cell migration and invasion by blocking Twist1-mediated epithelial-mesenchymal transition (EMT) (49). Although there are no previous studies on *DLL3* in uveal melanoma, *DLL4* was confirmed to be associated with the metastatic risk in uveal melanoma and its expression is the most inversely correlated with patient survival, which activates the NOTCH signaling pathway in uveal melanoma cells and controls their growth and migration (50).

Functional annotation including GO terms and KEGG pathways enrichment analyses revealed that DLL3 mainly participated in the regulation of the immune process. Setting the hallmarks of cancer as the reference gene sets, GSEA demonstrated that hallmarks such as epithelial-mesenchymal transition and signaling pathways like NOTCH signaling, KRAS signaling, and TNFα signaling *via* NF-κB were significantly enriched in the low *DLL3* expression group. Epithelial-mesenchymal transition is a process of cell remodeling during which epithelial cancer cells acquire migratory abilities (51). *KRAS* is the most frequently mutated oncogene and KRAS signaling functions as a main driver of tumorigenesis and development in various human cancers (52). Increasing evidence indicated that KRAS signaling could modulate the tumor microenvironment and promote tumor-related immune response (53), which was also found to be enriched in the low *DLL3* expression group. TNFα is a cytokine that mainly participates in inflammation and immune response, while in tumors, it’s reported that TNFα-NF-κB signaling plays a crucial role in promoting tumor cell migration and invasion (54). On the contrary, there were no hallmarks found in the high *DLL3* expression group. Taken together, it’s not surprising that UM patients with higher expression of *DLL3* were more likely to have good clinical outcomes.

There were also some limitations to our study. First, our study was mainly based on public retrospective datasets. Moreover, the sample size was limited and uneven sample sizes were between conjunctival melanoma and uveal melanoma. Conclusions need to be further verified by large sample studies. Besides, we used clinical data to verify the conclusions drawn from bioinformatics while the detailed molecular mechanism has not been elucidated. The relationship between DLL3 DNA methylation and DLL3 expression needs to be further validated by experiments.

In summary, this is the first study to identify that *DLL3*, a methylation-driven gene, may serve as a new potential diagnostic and prognostic biomarker in ocular melanoma. Our study may help improve the clinical outcomes of patients with UM or CM.

## Data availability statement

The original contributions presented in the study are included in the article/**Supplementary Material**. Further inquiries can be directed to the corresponding authors.

## Ethics statement

This research was performed in accordance with the World Medical Association Declaration of Helsinki. Written informed consent was obtained from all patients. The study was approved by the Ethics Committee of Shanghai JiaoTong University.

## Author contributions

LY and GW designed the study, analyzed data, and wrote the manuscript. SG, JR and RC designed the study and provided funding acquisition. LY, HS and SJ performed the experiments and analyzed the data. All authors read and approved the final submitted manuscript. All authors contributed to the article and approved the submitted version.

## Funding

This work was supported by the National Key Research and Development Plan (2017YFE0196300), the National Natural Science Foundation of China (No. U1932135 and No. 12275178), the Science and Technology Commission of Shanghai (20DZ2270800 and 22Y31900700), the Shanghai Pujiang Program (21PJD036), the Research project of Shanghai Municipal Health Commission (20204Y0302).

## Acknowledgments

The authors would like to thank the TCGA and GEO databases for the availability of the data.

## Conflict of interest

The authors declare that the research was conducted in the absence of any commercial or financial relationships that could be construed as a potential conflict of interest.

## Publisher’s note

All claims expressed in this article are solely those of the authors and do not necessarily represent those of their affiliated organizations, or those of the publisher, the editors and the reviewers. Any product that may be evaluated in this article, or claim that may be made by its manufacturer, is not guaranteed or endorsed by the publisher.

## Supplementary material

Supplementary Figure 1 The quality control analyses of samples from GSE57362. **(A)** PCA plot to visualize the relationship between UM and uvea samples. **(B)** Heatmap to exhibit the correlation among samples from GSE57362. **(C)** Heatmap to show the representative differential methylation CpGs between UM samples and normal uvea tissues.

Supplementary Figure 2 The correlation between the expression of DLL3 and the methylation level of its corresponding CpGs.

Supplementary Table 2 DEGs between metastatic UM samples and non-metastatic ones as well as DEGs between low and high DLL3 expression groups.

Supplementary Table 3 The correlation between the DEGs expression level and their DNA methylation level in UM. **P* < 0.05, ***P* < 0.01.

Supplementary Table 4 The methylation level of CpGs in *DLL3* was positively associated with UM.

Supplementary Table 5 The results of GO terms and KEGG pathways enrichment analyses as well as the results of GSEA of cancer hallmarks.
