# Supplementary material for: Methylation-driven gene DLL3 is a potential prognostic biomarker in ocular melanoma correlating with metastasis
Source: Front Oncol. 2022 Oct 20;12:964902. doi: 10.3389/fonc.2022.964902 (PMC9630341; doi:10.3389/fonc.2022.964902)
Supplement: Supplementary file 1 [file Table_1.docx]

**Additional file 1:**

**Table S1.** The clinical characteristics of ocular melanoma patient cohorts in tissue chip assay

| Features | Ocular melanoma | Normal mylanocyte |
| --- | --- | --- |
| Numbers | 60 | 18 |
| Gender, F/M | 17/43 | 12/6 |
| Age | 54.83±17.32 | 44.67±21.10 |
| Stage(AJCC) |  |  |
| T1 | 2 | / |
| T2 | 26 | / |
| T3 | 31 | / |
| T4 | 1 | / |
