# Supplementary figures and images for: Methylation-driven gene DLL3 is a potential prognostic biomarker in ocular melanoma correlating with metastasis
Source: Front Oncol. 2022 Oct 20;12:964902. doi: 10.3389/fonc.2022.964902 (PMC9630341; doi:10.3389/fonc.2022.964902)

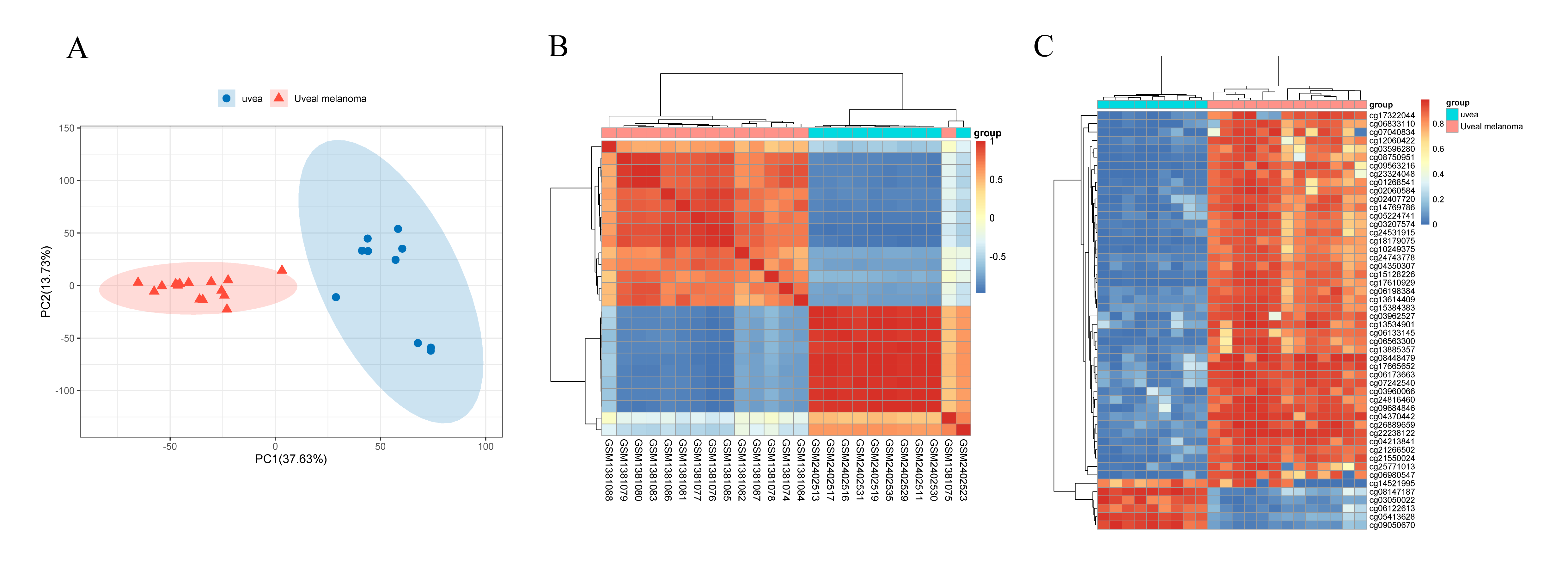

Supplement: Supplementary Figure 1 — The quality control analyses of samples from GSE57362. (A) PCA plot to visualize the relationship between UM and uvea samples. (B) Heatmap to exhibit the correlation among samples from GSE57362. (C) Heatmap to show the representative differential methylation CpGs between UM samples and normal uvea tissues. [file Image_1.tif]

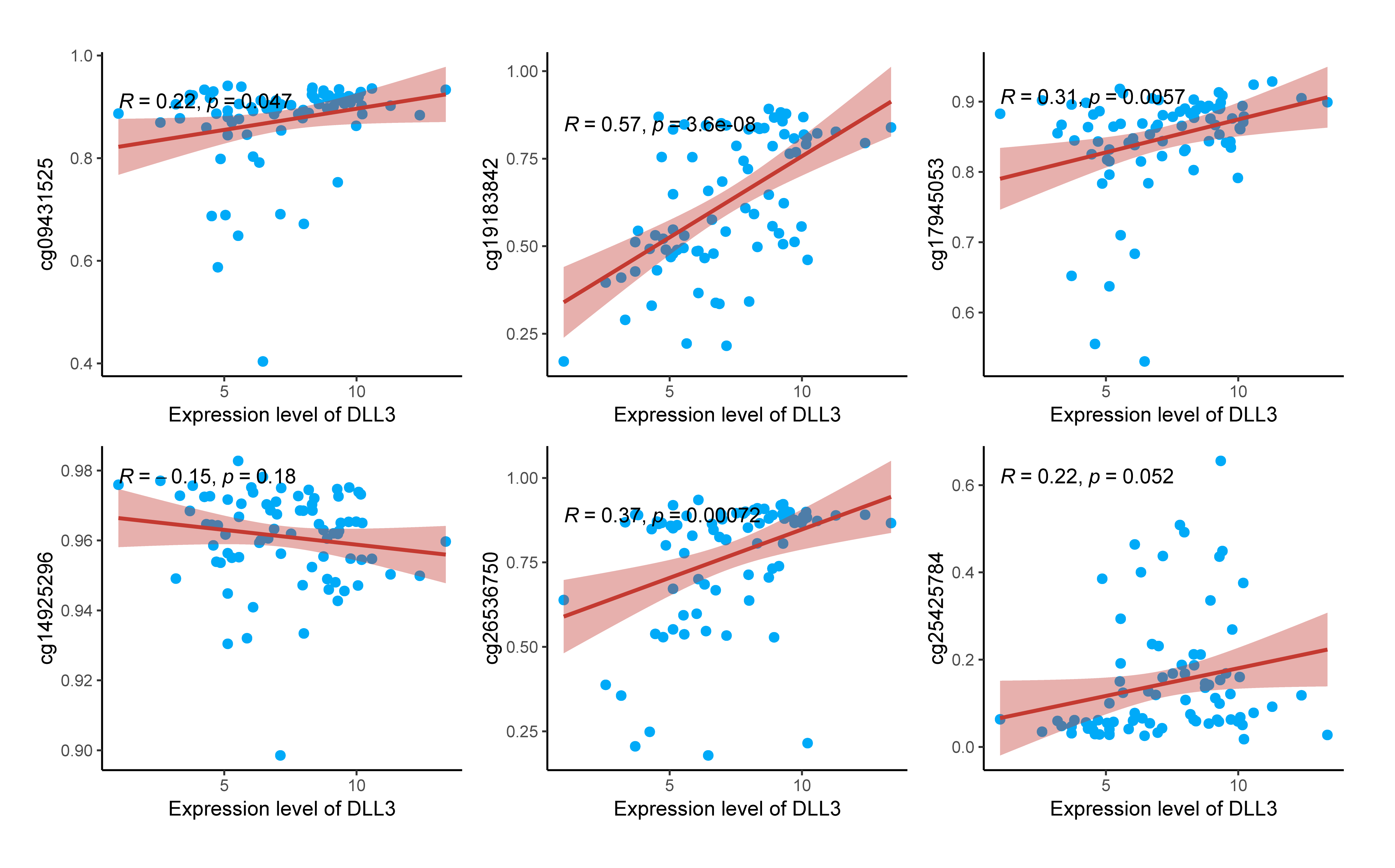

Supplement: Supplementary Figure 2 — The correlation between the expression of DLL3 and the methylation level of its corresponding CpGs. [file Image_2.tif]
